# Radiation Dose to Swallowing Muscles and Post-Radiotherapy Laryngeal Penetration or Aspiration in Head and Neck Squamous Cell Carcinoma

**DOI:** 10.3390/cancers18040543

**Published:** 2026-02-07

**Authors:** Thong Chotchutipan, Peesit Leelasawatsuk, Tiraya Phuengtrakul, Jinnatham Aphichato, Kemmapon Chumchuen, Jidapa Bridhikitti

**Affiliations:** 1 Department of Radiology, Faculty of Medicine, Prince of Songkla University, Songkhla 90110, Thailand; thong.chotchutipan@gmail.com (T.C.); jinnatham.aphichato@gmail.com (J.A.); 2 Department of Otolaryngology, Faculty of Medicine, Prince of Songkla University, Songkhla 90110, Thailand; peesit.l@psu.ac.th (P.L.); tiraya.ap@gmail.com (T.P.); 3 Department of Clinical Research and Medical Data Sciences, Faculty of Medicine, Prince of Songkla University, Songkhla 90110, Thailand; kemmapon.chumchuen@outlook.com

**Keywords:** swallowing, dysphagia, radiotherapy, head and neck cancer

## Abstract

Background/Objectives: Radiation-induced dysphagia can cause life-threatening complications including aspiration pneumonia. Consequently, studies have attempted to limit the radiation dose to the swallowing muscles to prevent radiation-induced dysphagia. However, data identifying the specific swallowing muscles associated with post-radiation dysphagia are lacking. Therefore, this case–control study aimed to identify the swallowing muscles whose radiotherapy-related damage is likely to cause radiation-induced dysphagia. Methods: This retrospective study included 53 patients with head and neck cancer who received definitive radiotherapy and underwent post-radiotherapy swallowing function evaluation using videofluoroscopy or fiberoptic endoscopic evaluation of swallowing at least 3 months post-radiotherapy. Twenty-two patients with a Penetration-Aspiration Scale score ≥ 3 were defined as having laryngeal penetration or aspiration. Twenty-two controls matched for age and tumor site were selected using propensity score matching. The primary exposure variable was radiation dose to the swallowing muscles, including the floor of the mouth, thyrohyoid, posterior digastric/stylohyoid, longitudinal pharynx, hyoglossus, styloglossus, genioglossus, and intrinsic tongue muscles. A LASSO regression model was used to select the most predictive dosimetric parameter. Results: The percentage of genioglossus muscle receiving a radiation dose ≥70 Gy (GGS V70) was the only dosimetric variable predictive of radiation-induced dysphagia. After adjusting for the clinical variables, GGS V70 demonstrated a significant association with post-radiotherapy laryngeal penetration or aspiration (*p* = 0.003), with an adjusted odds ratio of 1.06 for each increasing radiation dose unit of GGS V70. Conclusions: The genioglossus muscle might be associated with radiation-induced dysphagia and, therefore, should be further investigated in prospective studies.

## Simple Summary

Radiation-induced dysphagia is a common adverse effect of radiotherapy in patients with head and neck cancer and may lead to life-threatening complications, including aspiration pneumonia. This case–control study aimed to identify swallowing muscles whose radiation-related injury is associated with the development of radiation-induced dysphagia. In our cohort of 44 patients, the percentage of genioglossus muscle volume receiving a radiation dose ≥ 70 Gy (GGS V70) emerged as the dosimetric parameter predictive of radiation-induced dysphagia.

## 1. Introduction

Swallowing is a physiological process that transfers food from the oral cavity to the esophagus [1] and requires the intricate coordination of several normal organs in the head and neck region to smoothly transfer food while protecting the airway. Radiotherapy is a crucial component in the treatment of head and neck cancer; however, it can cause side effects, such as inflammation, edema, and fibrosis in these organs, leading to radiation-induced dysphagia. Accordingly, a study reported that aspiration pneumonia was detected in 21.3–62% of patients with head and neck cancer who received concurrent chemoradiotherapy [2,3,4]. Notably, patients who develop aspiration pneumonia after treatment have a higher mortality rate than those who do not [2].

Dysphagia/aspiration-associated structures (DARS) were first defined by Eisbruch et al. as organs whose damage from radiotherapy was likely to cause radiation-induced dysphagia [5]. They used videofluoroscopic swallowing studies (VFSS) and computed tomography (CT) scans to evaluate 26 patients with oropharyngeal cancer who received concurrent chemoradiotherapy and identified the pharyngeal constrictors, supraglottic, and glottic larynx as DARS. Several subsequent studies have also demonstrated an association between radiation dose to the pharyngeal constrictor muscle and larynx and radiation-induced dysphagia [6,7,8,9,10]. Consequently, current radiotherapy guidelines have included pharyngeal constrictors and the larynx as organs at risk for radiotherapy planning [11,12].

In recent years, researchers have been interested in adding muscles involved in the swallowing process to the DARS, with several studies showing an association between radiation dose to the swallowing muscles and radiation-induced dysphagia [13,14,15,16]. However, these studies were limited by variations in the definitions and selection of the evaluated swallowing muscles. Subsequently, the recent guidelines proposed by Gawryszuk et al. in a pioneering study provided comprehensive delineation guidelines using a CT-based atlas for the muscles involved in the swallowing process [1,17]. These guidelines have standardized the definition of swallowing muscles; however, data identifying the specific swallowing muscles associated with post-radiation dysphagia are lacking. Therefore, this study aimed to identify the swallowing muscles whose radiotherapy-related damage is likely to cause radiation-induced dysphagia, defined as post-radiation laryngeal penetration or aspiration. Additionally, this study sought to explore the radiation dose constraints on the swallowing muscles associated with post-radiation laryngeal penetration or aspiration.

## 2. Materials and Methods

### 2.1. Study Population

We retrospectively reviewed the data of patients who received radiotherapy at Songklanagarind Hospital, Thailand, between January 2014 and December 2024. Patients were included in this study if they met the following criteria: (1) had a histologically confirmed diagnosis of previously untreated squamous cell carcinoma of the head and neck; (2) aged ≥ 18 years; (3) received definitive radiotherapy using intensity-modulated radiotherapy technique with or without chemotherapy; (4) underwent a post-radiotherapy swallowing function assessment using VFSS or fiberoptic endoscopic evaluation of swallowing (FEES) at least 3 months after radiotherapy; and (5) were cancer-free at the time of VFSS or FEES. Patients were excluded from the study if they met any of the following criteria: (1) diagnosed with laryngeal, hypopharyngeal, or oral cavity cancers; (2) underwent radical surgery prior to radiotherapy; (3) had synchronous secondary cancer; or (4) had distant metastasis at the time of diagnosis. Additionally, patients whose radiotherapy plans could not be retrieved from the radiotherapy planning system were excluded. Patients with laryngeal and hypopharyngeal cancers were excluded because these subsites typically receive high radiation doses to the larynx during treatment, which could confound the assessment of radiation-induced dysphagia. Patients with oral cavity cancer were also excluded, as they were primarily treated with upfront surgery, which may directly affect swallowing muscles. The study was approved by the Human Research Ethics Committee, Faculty of Medicine, Prince of Songkla University (REC.67-531-7) and the requirement for informed consent was waived due to the retrospective nature of the study.

### 2.2. Radiotherapy Treatment

Radiotherapy planning was conducted using the Eclipse Treatment Planning System (v.16.0; Varian Medical Systems, Palo Alto, CA, USA). Target volume delineation was performed using contrast-enhanced CT simulation. Gross tumor volume (GTV) was defined as the primary tumor and involved lymph nodes. The high-risk clinical target volume (CTV-HR) was defined as the GTV plus a 5–10 mm margin, modified to anatomical barriers. The intermediate-risk CTV (CTV-IR) was defined as CTV-HR plus a 5 mm margin and extended to encompass the involved nodal regions. In nasopharyngeal cancer, CTV-IR also included the parapharyngeal space, clivus, sphenoid sinus, posterior third of the nasal cavity, and maxillary sinuses. The elective clinical target volume included nodal levels considered at risk of microscopic disease based on the primary tumor site and stage. Planning target volumes (PTVs) were generated by adding a 5 mm isotropic margin to each CTV. The dose prescription was as follows: 70 Gy in 33–35 fractions to the PTV-HR, 59.4–63 Gy in 33–35 fractions to the PTV-IR, and 54–56 Gy in 33–35 fractions to the PTV-EL, using simultaneous integrated boost.

### 2.3. Swallowing Assessment

Swallowing function assessments were performed by an otolaryngologist and a speech pathologist, each with more than 5 years of clinical experience in swallowing disorders. FEES was typically the first-line modality owing to its availability and ease of access in our institution. However, VFSS was performed in cases where additional information was deemed necessary or when the examining physician and speech pathologist considered VFSS to provide more comprehensive diagnostic data. Therefore, the selection between FEES and VFSS was guided by both clinical indications and resource availability.

The consistencies and volumes used for routine clinical examinations included 3, 5, and 10 mL of mildly thick and thin liquids. Each bolus was swallowed in a single attempt, with suction available at the bedside. Patients were instructed to retain the bolus in their mouth until they were prompted to swallow. For safety reasons, the administered volumes were adjusted in cases where there was a risk of aspiration, based on clinical symptoms. If aspiration was observed (Penetration-Aspiration Scale [PAS] scores 6–8), the otolaryngologist and speech-language pathologist determined whether additional trials with larger volumes or alternative consistencies posed an excessive risk. When such a risk was identified, the examination was discontinued at the safest consistency and volume tolerated by the patient. Penetration and aspiration events were scored using the PAS score by consensus between the otolaryngologist and speech pathologist.

FEES was conducted in the clinic with the participants seated in an upright position. A flexible nasoendoscope was inserted transnasally and advanced to the pharynx to obtain a superior view of the laryngopharynx, with the tip positioned just above the epiglottis at a high position. All examinations were video-recorded and stored on a local drive. No nasal vasoconstrictor or topical anesthetic was applied to the nasal mucosa before the procedure.

VFSS was performed with the patients seated upright in the lateral projection, with an anteroposterior view obtained as needed. Continuous fluoroscopy at 30 frames per second was used to record swallowing from the oral preparatory phase through the esophageal phase. Test boluses were administered with an interval of 30–60 s between swallows.

### 2.4. Case Group

Cases were defined as patients who exhibited post-radiation laryngeal penetration or aspiration detected via VFSS or FEES conducted at least 3 months after radiotherapy. This time frame was selected because it is conventionally used to differentiate acute from late radiation effects [18]. Acute radiation adverse events were defined as those occurring from the initiation of radiotherapy up to 90 days thereafter, whereas late radiation adverse events were defined as those occurring more than 90 days after the start of radiotherapy. Accordingly, swallowing assessments performed at least 3 months after radiotherapy were expected to minimize the influence of acute inflammatory reactions and more accurately reflect late radiation-induced dysphagia. VFSS and FEES examinations were retrospectively reviewed by an otolaryngologist and scored using the PAS. A PAS score ≥ 3 was used to define post-radiation laryngeal penetration or aspiration [19,20,21]. Data assignment and statistical analysis were conducted independently. The otolaryngologist was blinded to all radiation dose–volume parameters throughout the scoring process.

### 2.5. Control Group

Patients without post-radiation laryngeal penetration or aspiration in the same database as the case group were eligible as controls. Once the case data were obtained, control selection from the control pool was conducted using propensity score matching with the MatchIt R package (version 4.7.2) [22]. Matching variables included age and tumor site, as both variables have a confounding effect on post-radiation dysphagia [23,24]. A ratio of 1 to the nearest score was set using a caliper width of 0.25. The balance between matching variables was assessed using the absolute standardized mean difference, along with the univariate association results of the matching variables between the two outcome groups.

### 2.6. Variables

#### 2.6.1. Exposure

**Figure 1:**
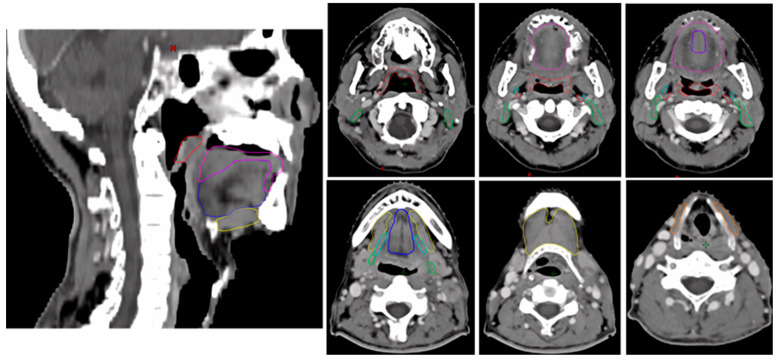
Example of swallowing muscle delineation. The figure illustrates the floor of the mouth muscle (FOM; yellow), genioglossus muscle (GGS; blue), intrinsic tongue muscles (ITM; magenta), posterior digastric/stylohyoid muscle complex (PDS; green), longitudinal pharyngeal muscles (LPM; red), hyoglossus/styloglossus muscle complex (HSG; cyan), and thyrohyoid muscle (THM; orange).

The primary exposure variable was the radiation dose to the swallowing muscles. The swallowing muscles included the floor of the mouth (FOM), thyrohyoid muscle, posterior digastric/stylohyoid muscle complex, longitudinal pharyngeal muscles (LPM), hyoglossus/styloglossus muscle complex (HSG), genioglossus muscle (GGS), and intrinsic tongue muscles (ITM). The muscles were defined based on the guidelines proposed by Gawryszuk et al. [1,17] and were retrospectively delineated in the CT simulations of patients by radiation oncologists (TC and JB) using the Eclipse Treatment Planning System (v.16.0; Varian Medical Systems, Palo Alto, CA, USA). Figure 1 shows an example of swallowing muscle delineation.

Dose-volume histograms for each swallowing muscle were calculated using the Eclipse Treatment Planning System v.16.0. The dosimetric values of interest in this study were the minimum, mean, and maximum radiation doses delivered to the swallowing muscles. In addition, the percentage volume of each swallowing muscle that received at least 30, 40, 50, 60, and 70 Gy (V30, V40, V50, V60, and V70, respectively) was recorded.

#### 2.6.2. Outcome

The primary outcome of this study was post-radiation laryngeal penetration or aspiration, as detected via VFSS or FEES, conducted at least 3 months after radiotherapy. As described above, a PAS score of ≥3 was used to define post-radiation laryngeal penetration or aspiration [19,20,21].

### 2.7. Confounders

#### 2.7.1. Clinical Confounders

Clinical confounders were selected based on previous studies that demonstrated their association with post-radiation dysphagia, as well as variables showing an association with the outcome in the univariate analysis (*p* < 0.1). Based on prior evidence [15,23,24,25,26,27], the following variables were included: age at the time of radiotherapy, pre-treatment performance and nutritional status, tumor stage and site, presence of tracheostomy, chemotherapy use, and receiving swallowing rehabilitation. These clinical confounders were retrospectively assessed using the in-house Hospital Information System (version 1) at Songklanagarind Hospital, Thailand. Pre-treatment nutritional status was assessed using the Subjective Global Assessment [28].

#### 2.7.2. Dosimetric Confounders

Dosimetric confounders included the mean radiation dose to the superior, middle, and inferior pharyngeal constrictor muscles, cricopharyngeal muscle, and supraglottic and glottic larynx [6,7,8,9,10]. These organs were retrospectively delineated in the CT simulations of the patients by radiation oncologists (TC and JB) using the Eclipse Treatment Planning System v.16.0. in accordance with the guidelines proposed by Brouwer et al. [29]. The mean radiation dose delivered to these organs was calculated using the Eclipse Treatment Planning System v.16.0.

### 2.8. Statistical Analysis

All collected variables are summarized using descriptive statistics. Continuous variables are presented as mean and standard deviation or median and interquartile range, depending on their distribution. Categorical variables are presented as frequencies and percentages. Univariate association tests were performed between the two outcome groups to describe differences in the variables. Variable selection for the multivariate analysis was performed based on the type of independent variable. A least absolute shrinkage and selection operator (LASSO) regression model was used to reduce the dimensions of the dosimetric variables while increasing the generalizability of the results. We created a full model consisting of 62 dosimetric variables to predict the outcome variable as an input for LASSO regression. The minimum lambda was used to specify the extent of shrinkage of the coefficients of the variables. Along with LASSO regression, the minimum lambda was extracted using the function *cv.glm* from the *glmnet* R package (version 4.1-10) [30]. The minimum value was selected from the lambda that yielded the lowest average prediction error in the 10-fold cross-validation process running internally in the function. Variables with unshrunken coefficients were used for further analysis. In contrast, the independent clinical variables were selected based on previous studies that demonstrated their association with post-radiation dysphagia, as well as variables showing an association with the outcome in the univariate analysis (*p* < 0.1). Age at radiotherapy, performance status, pre-treatment nutritional status, presence of tracheostomy, chemotherapy use, tumor site, tumor stage, and receiving swallowing rehabilitation were selected in the multivariate model based on previous evidence of their association with outcome variables [15,23,24,25,26,27]. The final model consisted of the remaining dosimetric and clinical variables, which were fitted to a multiple logistic regression model, variables found with high VIF score representing multicollinearity were reviewed and excluded from the final model. The results are presented as odds ratios (ORs) and 95% confidence intervals (CIs). Dosimetric variables with significantly adjusted OR were further explored for their association with outcome status using the receiver operating characteristic (ROC) curve to investigate their explanatory power in predicting the outcome. While we did not implement bootstrapping during the model selection process to prevent potential complete separation of variables within resampled datasets, we instead applied bootstrapping to assess the stability of the final model containing the selected dosimetric variable. Comparisons between the model trained on the full training dataset and models generated using bootstrap resampling are presented using ROC curves, along with other performance metrics, to evaluate consistency of results. Furthermore, the performance metrics were also accompanied by 95% CIs through the mean of bootstrapping predictions from the prior bootstrap process for model validation. Statistical analysis was performed using the R program version 4.5.1 [31]. Statistical significance was defined as a *p*-value < 0.05.

## 3. Results

Of the 53 eligible patients, 22 were classified into the case group and 31 into the control group. Propensity score matching resulted in 22 matched case–control pairs. The distribution of matching variables between the groups was well-balanced (Table S1).

Most patients (81.8%) were men with a mean age of 53.5 years. The majority (91%) of the patients had a history of smoking. Approximately two-thirds of the patients had nasopharyngeal tumors. Most patients had locally advanced disease, with 29.5% at Stage III and 68.2% at Stage IV. The median radiotherapy dose was 70 Gy (range, 64–70 Gy). All patients received chemotherapy, with 56.8% receiving concurrent and adjuvant chemotherapy, 29.5% receiving concurrent chemotherapy, and 13.6% receiving induction and concurrent chemotherapy. Univariate analysis showed no statistical differences in any descriptive characteristics between the two groups (Table 1).

The radiation dosimetric parameters are summarized in Table 2, which reports the mean, minimum, and maximum doses (Gy) delivered to each swallowing muscle. In addition, the percentage volume of each swallowing muscle receiving at least 30, 40, 50, 60, and 70 Gy (V30, V40, V50, V60, and V70, respectively) is reported.

**Figure 2:**
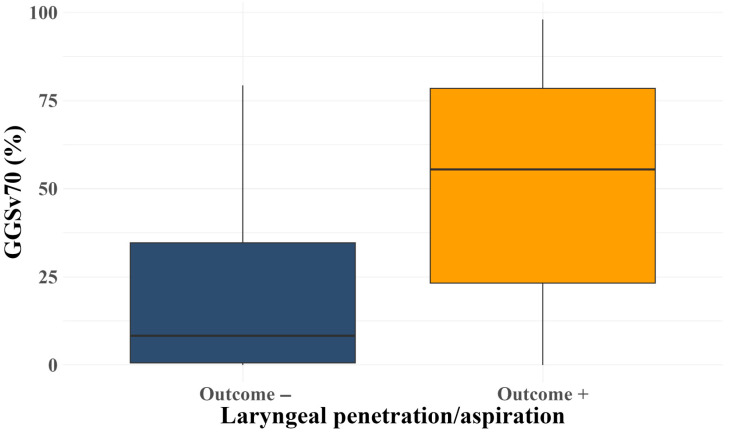
Boxplots of GGS V70 with post-radiotherapy penetration or aspiration. GGS V70: genioglossus muscle receiving a radiation dose ≥ 70 Gy.

LASSO regression performed using the minimum lambda of 0.153 identified the percentage of the GGS muscle receiving radiation dose ≥ 70 Gy (GGS V70) as the only predictive factor. The distribution of GGS V70 between the two outcome groups is shown in Figure 2. A higher distribution of GGS V70 was observed in the laryngeal penetration and aspiration groups. After adjusting for clinical confounding variables, GGS V70 demonstrated a significant association with post-radiotherapy laryngeal penetration or aspiration (p_wald’s_ = 0.003), with an adjusted OR of 1.06 for each increasing unit of GGS V70 (Table 3).

**Figure 3:**
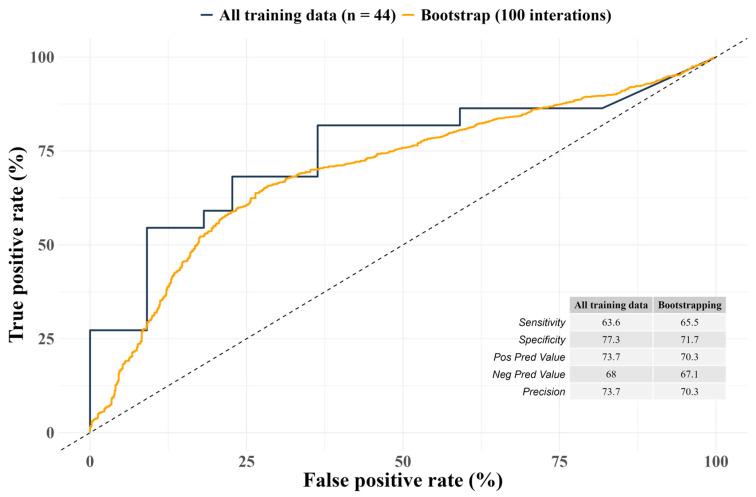
Receiver operating characteristic curves of GGS V70 against post-radiotherapy aspiration or penetration.

Regarding the association between GGS V70 and post-radiotherapy aspiration or penetration, receiver operating characteristic curve with only GGS V70 as the sole predictor gave an accuracy of 70.5% as the detected area under the curve, the respective metric from bootstrap was 68.6% (95% CI: 66.2, 70.8). The curves along with other performance metrics are displayed in Figure 3. Overall, the performance of the bootstrap process fell slightly behind (specificity of 71.7%, 95% CI: 68.7, 74.3; positive predictive value of 70.3, 95% CI: 67.2, 73.5; negative predictive value of 67.1, 95% CI: 63.8, 70.2), except the model sensitivity (65.5%, 95% CI: 62.3, 68.9), which was slightly higher.

## 4. Discussion

This case–control study aimed to identify the swallowing muscles whose damage from radiotherapy is likely to cause radiation-induced dysphagia. The results of LASSO regression suggested that the percentage of GGS V70 was significantly associated with post-radiotherapy laryngeal penetration or aspiration. However, given the relatively small sample size, this association should be interpreted as exploratory and hypothesis-generating.

The GGS is an extrinsic muscle of the tongue, with two muscular compartments: oblique and horizontal, that plays an important role in swallowing [32,33]. The oblique compartment originates in the mandible and expands in a fan-like manner into the intrinsic tongue muscle, thereby contributing to the preparatory and oral phases of swallowing. The horizontal compartment arises from the mandible and is inserted into the hyoid bone and the base of the tongue. During the pharyngeal phase of swallowing, the horizontal compartment pulls the hyoid and larynx forward, protecting the airway from food aspiration and assisting in opening the pharynx [32]. Pu et al. previously reported that a higher radiation dose to the GGS muscle was significantly associated with shorter processing times during all swallowing phases in 44 patients with nasopharyngeal carcinoma [34]. This may reflect a disruption in normal swallowing coordination or premature bolus movement, potentially increasing the aspiration risk.

Kumar et al. retrospectively reviewed the data of 46 patients with oropharyngeal cancer who received concurrent chemoradiotherapy. Defining radiation-induced dysphagia as a PAS score ≥ 3, their multivariate model showed a significant association between radiation dose to the FOM muscle with radiation-induced dysphagia [14]. Similarly, Dale et al. explored the association between radiation dose to the swallowing muscles and chronic radiation-induced dysphagia in 300 patients with oropharyngeal cancer [16]. They defined chronic radiation-induced dysphagia as the presence of aspiration or stricture detected on videofluoroscopy or endoscopy, gastrostomy dependence, and/or aspiration pneumonia occurring 1 year or more after radiotherapy. Using recursive partitioning analysis, the study found that the radiation dose to the FOM muscle (defined as the mylo/geniohyoid complex), GGS muscle, anterior digastric muscle, and superior and middle constrictor muscles was associated with chronic radiation-induced dysphagia. A multivariate model with clinical parameters demonstrated that radiation dose to the FOM muscle and age were the most predictive variables [16]. Thus, our study is consistent with previous studies that have demonstrated an association between radiation dose to the GGS muscle and post-radiation dysphagia. However, while previous studies have identified radiation dose to the FOM muscle as the most predictive factor for radiation-induced dysphagia, our analysis found that radiation dose to the GGS muscle was the strongest predictor. This discrepancy may be attributed to the higher radiation dose to the FOM muscles observed in our study which may have attenuated the ability of dosimetric parameters of the FOM muscle to discriminate between cases and controls. Furthermore, variations in the definitions of swallowing muscles across studies may have contributed to the different results.

Compared with traditional mean dose constraints for the pharyngeal constrictor muscles, our findings appear to indicate that high-dose volume metrics may better capture the risk of radiation-related dysphagia, potentially reflecting localized tissue injury that is not fully represented by mean dose alone. Supporting this concept, Dale et al. demonstrated that the percentage of the FOM muscle receiving radiation dose ≥ 69 Gy (FOM V69) was significantly associated with chronic radiation-induced dysphagia [16]. In contrast, other studies have reported associations between mean radiation dose to swallowing muscles and dysphagia [10,14], suggesting that both global and focal dose effects may contribute to swallowing dysfunction.

From a practical standpoint, the feasibility of applying a GGS dose constraint must be considered in the context of competing priorities in head and neck IMRT planning. The GGS is anatomically adjacent to high-dose target volumes in many oral cavity and oropharyngeal tumors, so attempts to reduce radiation dose to the GGS may trade off against target coverage. In clinical practice, we therefore envision GGS dose constraint being used as a planning objective or soft constraint, rather than a rigid numerical limit, with dosimetrist seeking to minimize the high-dose volume to the GGS while preserving adequate PTV coverage. For tumors in which the high-dose PTV does not directly involve the GGS, meaningful reductions in radiation dose to the GGS may be achievable through modest adjustments in beam arrangement and optimization priorities.

One of the main limitations of our study was its retrospective nature, which could have caused a selection bias. Patients who underwent VFSS or FEES were more likely to be at a higher risk of radiation-induced dysphagia. Additionally, VFSS or FEES were not performed at a uniform time point for all patients. This temporal heterogeneity may introduce misclassification and time-related confounding, as swallowing function can both recover in the subacute phase and deteriorate over time due to progressive radiation-induced fibrosis. We attempted to address this limitation by including the time from radiotherapy to swallowing assessment in the multivariate analysis to reduce potential bias. However, in this retrospective cohort, swallowing assessments were performed as part of routine clinical care based on symptom presentation rather than at standardized time points. Pooling subacute and late post-treatment assessments therefore reflects real-world practice and allowed inclusion of all available, clinically relevant evaluations.

Furthermore, swallowing outcomes (PAS score) were assessed using either FEES or VFSS, which differ in their sensitivity for detecting penetration and aspiration. However, PAS scoring for both modalities was performed by a single experienced otolaryngologist blinded to all dosimetric data, minimizing differential bias. The Penetration–Aspiration Scale has been validated for use with both FEES and VFSS and demonstrates good reliability [35]. Combining data from both modalities increased statistical power and reflects real-world clinical practice in head and neck cancer survivorship. Nevertheless, intra-rater reliability was not formally assessed, which may introduce measurement variability, although the use of standardized PAS criteria by a single rater may have mitigated this effect. Another limitation was the heterogeneity of the primary cancer in this study. Patients with different primary cancers received different radiation exposures. Patients with oropharyngeal cancer typically receive higher radiation doses to swallowing muscles in the oral cavity, including FOM, ITM, GGS, and HSG, whereas patients with nasopharyngeal cancer receive comparatively higher doses to pharyngeal muscles, particularly the LPM. To account for these systematic differences in treatment fields and dose distribution, we performed propensity score matching between the case and control cohorts based on the primary tumor site, thereby minimizing bias and reducing potential confounding effects when evaluating swallowing outcomes.

The other main limitation was the limited sample size. While we obtained all eligible cases from the study sites, the number of cases with laryngeal penetration or aspiration were only a few. This could lead to the problem of model instability using due to the aspect like low events-per-variable. We addressed the events-per-variable limitation through only including one dosimetric predictor for the prediction model, which was GGS V70. Moreover, to prevent overclaiming model performance in case of model instability, bootstrapping with 100 iterations was conducted for sensitivity analysis. The bootstrap result, while slightly lower in performance matrixes, coincided with those predicting the trained data suggesting that the model was stable to an extent.

It should be noted that the overall significant association between GGS and laryngeal penetration or aspiration found in our study with a limited sample size was model-dependent. While removing variables such as chemotherapy sequence and swallowing rehabilitation due to their collinearity may have improved the accuracy of model estimation parameters, such variables were also clinically relevant. Consequently, residual confounding cannot be excluded, and the findings regarding GGS V70 should therefore be interpreted with caution. Furthermore, although time to swallowing assessment was included as a covariate in the analysis, the retrospective and symptom-driven timing of swallowing evaluations limits our ability to draw conclusions regarding the temporal evolution or causality of radiation-related swallowing dysfunction.

Our findings should be interpreted in the context of the outcome definition. Specifically, the observed associations pertain to laryngeal penetration and/or aspiration events, as defined by the PAS score, rather than to aspiration alone. Accordingly, these results should not be extrapolated to more severe dysphagia outcomes. Additionally, the proposed GGS V70 probability threshold should be regarded as exploratory, rather than an established or clinically validated planning constraint.

Finally, our cohort was predominantly composed of patients with nasopharyngeal carcinoma treated at a single institution, which may limit the generalizability of our findings to other head and neck subsites or treatment settings. Nevertheless, as swallowing musculature and the biological mechanisms of radiation-related injury are largely shared across head and neck subsites, these findings may still provide hypothesis-generating insights relevant to broader head and neck cancer populations. Moreover, the extended study period may have introduced temporal heterogeneity due to evolving radiotherapy protocols, dose constraints, and supportive care practices. Although all patients received definitive intensity-modulated radiotherapy, limiting major technique-related variability, more subtle temporal changes cannot be excluded and should be considered when interpreting the results. Further validation in multi-institutional cohorts with more disease sites is warranted.

## 5. Conclusions

Our study suggests that the GGS muscle is associated with radiation-induced dysphagia. However, given the relatively small sample size, this observation should be interpreted as exploratory and hypothesis-generating, and requires further validation in larger, independent cohorts before consideration for clinical implementation. Future studies should analyze the two compartments of the GGS muscle as different structures because they have different functions in the swallowing process. Consequently, studies are required to identify the compartment that contributes to post-radiation dysphagia.

## Figures and Tables

**Table 1:** Descriptive characteristics between patients with and without post-radiotherapy laryngeal penetration or aspiration.

Variable	Total	Without Laryngeal Penetration/Aspiration	With Laryngeal Penetration/Aspiration	*p*-Value
	(*n* = 44)	(*n* = 22)	(*n* = 22)	
PAS				<0.001 ^b^
Median (IQR)	2.5 (1, 4)	1 (1, 2)	4 (3, 7)	
Age				0.119 ^a^
Mean (SD)	53.5 (9.5)	55.7 (8.3)	51.2 (10.2)	
Swallowing assessment				0.125 ^c^
FEES	18 (40.9)	6 (27.3)	12 (54.5)	
VFSS	26 (59.1)	16 (72.7)	10 (45.5)	
Time from radiotherapy to swallowing assessment (months)				0.071 ^b^
Median (IQR)	10.8 (8, 20.1)	9.6 (6.9, 14.8)	14.6 (8.8, 29.4)	
Sex				0.24 ^d^
Male	36 (81.8)	16 (72.7)	20 (90.9)	
Female	8 (18.2)	6 (27.3)	2 (9.1)	
Site				1 ^c^
Nasopharynx	30 (68.2)	15 (68.2)	15 (68.2)	
Oropharynx	14 (31.8)	7 (31.8)	7 (31.8)	
ECOG PS				1 ^d^
0	1 (2.3)	0 (0)	1 (4.5)	
1	2 (4.5)	1 (4.5)	1 (4.5)	
2	40 (90.9)	20 (90.9)	20 (90.9)	
3	1 (2.3)	1 (4.5)	0 (0)	
SGA				0.894 ^d^
A	27 (61.4)	14 (63.6)	13 (59.1)	
B	12 (27.3)	5 (22.7)	7 (31.8)	
C	1 (2.3)	1 (4.5)	0 (0)	
Missing	4 (9.1)	2 (9.1)	2 (9.1)	
Smoking				0.108 ^d^
No	3 (6.8)	3 (13.6)	0 (0)	
Yes	40 (90.9)	18 (81.8)	22 (100)	
Missing	1 (2.3)	1 (4.5)	0 (0)	
Feeding tube during treatment				0.488 ^d^
No	2 (4.5)	0 (0)	2 (9.1)	
Yes	42 (95.5)	22 (100)	20 (90.9)	
Tracheostomy				0.233 ^d^
No	41 (93.2)	22 (100)	19 (86.4)	
Yes	3 (6.8)	0 (0)	3 (13.6)	
T stage				0.864 ^d^
1	1 (2.3)	0 (0)	1 (4.5)	
2	2 (4.5)	1 (4.5)	1 (4.5)	
3	11 (25)	5 (22.7)	6 (27.3)	
4	30 (68.2)	16 (72.7)	14 (63.6)	
N stage				0.265 ^d^
0	1 (2.3)	0 (0)	1 (4.5)	
1	1 (2.3)	0 (0)	1 (4.5)	
2	27 (61.4)	16 (72.7)	11 (50)	
3	15 (34.1)	6 (27.3)	9 (40.9)	
Stage group				0.51 ^c^
II	1 (2.3)	1 (4.5)	0 (0)	
III	13 (29.5)	5 (22.7)	8 (36.4)	
IV	30 (68.2)	16 (72.7)	14 (63.6)	
Chemotherapy sequence				0.196 ^d^
Concurrent	13 (29.5)	5 (22.7)	8 (36.4)	
Induction and concurrent	6 (13.6)	5 (22.7)	1 (4.5)	
Concurrent and adjuvant	25 (56.8)	12 (54.5)	13 (59.1)	
Radiation dose				0.34 ^b^
Median (IQR)	70 (70, 70)	70 (70, 70)	70 (70, 70)	
Swallowing rehabilitation				1 ^c^
Yes	17 (38.6)	9 (40.9)	8 (36.4)	
No	27 (61.4)	13 (59.1)	14 (63.6)	

^a^ Student’s *t*-test; ^b^ Mann–Whitney *U* test; ^c^ chi-squared test; ^d^ Fisher’s exact test. PAS: Penetration-Aspiration Scale, ECOG PS: Eastern Cooperative Oncology Group Performance Status, SGA: Subjective Global Assessment, IQR: interquartile range, SD: standard deviation, FEES: fiberoptic endoscopic evaluation of swallowing, VFSS: videofluoroscopic swallowing studies.

**Table 2:** Dosimetric parameters of swallowing muscles per laryngeal penetration/aspiration status.

Variable	Total	Without Laryngeal Penetration/Aspiration	With Laryngeal Penetration/Aspiration	*p*-Value
	(*n* = 44)	(*n* = 22)	(*n* = 22)	
FOMmean	69.99 (64.28, 73.82)	69.5 (61, 71)	72.9 (67.6, 74.7)	0.017 ^b^
FOMmin	52.6 (12.6)	50.1 (10.6)	55 (14.1)	0.199 ^a^
FOMmax	77.35 (75.06, 79.23)	76.3 (74.1, 77.8)	78.9 (75.9, 82.8)	0.009 ^b^
FOM V30	100 (100, 100)	100 (100, 100)	100 (100, 100)	0.34 ^b^
FOM V40	100 (100, 100)	100 (100, 100)	100 (100, 100)	0.754 ^b^
FOM V50	100 (98.31, 100)	100 (96.4, 100)	100 (100, 100)	0.392 ^b^
FOM V60	97.88 (81.42, 100.00)	96.9 (65.6, 99)	99.3 (90, 100)	0.157 ^b^
FOM V70	62.66 (9.39, 93.79)	51.9 (7.9, 69.4)	89.7 (40, 99.9)	0.029 ^b^
THMmean	67.1 (8.7)	64.5 (7.7)	69.6 (9)	0.049 ^a^
THMmin	45.7 (14.4)	43.3 (13.7)	48.1 (14.9)	0.271 ^a^
THMmax	76.8 (5.7)	74.8 (4.4)	78.8 (6.2)	0.018 ^a^
THM V30	100 (100, 100)	100 (100, 100)	100 (100, 100)	0.655 ^b^
THM V40	100 (99.92, 100)	100 (99.9, 100)	100 (100, 100)	0.59 ^b^
THM V50	99.97 (95.32, 100)	98.5 (94.2, 100)	100 (99.3, 100)	0.098 ^b^
THM V60	94.62 (70.84, 99.74)	77.4 (64.3, 97.6)	99.5 (89, 100)	0.048 ^b^
THM V70	46.1 (37.7)	32.8 (36.4)	59.3 (35)	0.018 ^a^
PDSmean	71.4 (5)	69.8 (3.8)	72.9 (5.6)	0.038 ^a^
PDSmin	49.7 (14.1)	46 (14.9)	53.5 (12.6)	0.078 ^a^
PDSmax	76.93 (75.87, 78.29)	76.5 (75.4, 77.3)	78 (76.2, 80.5)	0.042 ^b^
PDS V30	100 (100, 100)	100 (100, 100)	100 (100, 100)	0.081 ^b^
PDS V40	100 (100, 100)	100 (99.7, 100)	100 (100, 100)	0.141 ^b^
PDS V50	99.98 (98.09, 100)	99.5 (97.2, 100)	100 (99.7, 100)	0.103 ^b^
PDS V60	97.59 (89.65, 100)	94.3 (87.5, 99.8)	99.3 (92, 100)	0.227 ^b^
PDS V70	78.59 (59.81, 96.43)	68.2 (55.3, 88.2)	87.1 (70.2, 98.5)	0.139 ^b^
LPMmean	73.42 (72.12, 75.08)	73.3 (71.3, 74.3)	73.7 (72.3, 75.8)	0.105 ^b^
LPMmin	68.99 (60.06, 71.32)	69.2 (61.4, 71)	68.1 (59.8, 71.9)	0.778 ^b^
LPMmax	76.80 (75.39, 78.34)	76.6 (75.2, 77.6)	77.3 (76.3, 80.4)	0.156 ^b^
LPM V30	100 (100, 100)	100 (100, 100)	100 (100, 100)	0.162 ^b^
LPM V40	100 (100, 100)	100 (100, 100)	100 (100, 100)	0.081 ^b^
LPM V50	100 (100, 100)	100 (100, 100)	100 (100, 100)	0.279 ^b^
LPM V60	100 (100, 100)	100 (100, 100)	100 (100, 100)	0.914 ^b^
LPM V70	99.78 (87.16, 100)	99.9 (80.9, 100)	98.9 (89.9, 100)	0.942 ^b^
HSGmean	72.25 (67.30, 74.00)	69.4 (65.3, 72.9)	73.5 (71.8, 75.9)	0.007 ^b^
HSGmin	59.16 (49.55, 66.26)	54.9 (45.7, 59.4)	65.4 (56.8, 69.3)	0.012 ^b^
HSGmax	76.50 (74.77, 78.28)	75.8 (74.5, 77.3)	77.7 (76.1, 79.8)	0.032 ^b^
HSG V30	100 (100, 100)	100 (100, 100)	100 (100, 100)	0.34 ^b^
HSG V40	100 (100, 100)	100 (100, 100)	100 (100, 100)	0.537 ^b^
HSG V50	100 (99.97, 100)	100 (98.4, 100)	100 (100, 100)	0.134 ^b^
HSG V60	99.91 (88.32, 100)	99.1 (76.6, 99.9)	100 (99.8, 100)	0.008 ^b^
HSG V70	83.85 (39.59, 99.09)	64.5 (35.6, 90.3)	98.3 (80.9, 99.8)	0.028 ^b^
GGSmean	62.82 (54.45, 67.66)	57.4 (52.9, 62.7)	65.4 (62.8, 71.9)	0.004 ^b^
GGSmin	35.6 (10)	32.7 (6.7)	38.6 (12)	0.049 ^a^
GGSmax	75.02 (72.64, 77.84)	74.4 (71.9, 77)	76.6 (74.2, 80.6)	0.069 ^b^
GGS V30	100 (99.89, 100)	100 (99.5, 100)	100 (100, 100)	0.482 ^b^
GGS V40	95.41 (88.49, 100)	91.9 (84.2, 98.2)	98.3 (94, 100)	0.041 ^b^
GGS V50	82.20 (67.64, 96.20)	76.5 (58.6, 87.3)	86.2 (81.7, 99.9)	0.011 ^b^
GGS V60	58.1 (32.1)	46.1 (28.8)	70.1 (31.3)	0.012 ^a^
GGS V70	35.7 (33.3)	20.5 (25.5)	51 (33.7)	0.002 ^a^
ITMmean	56.4 (9.3)	54.3 (7)	58.6 (10.9)	0.122 ^a^
ITMmin	30.57 (27.48, 36.65)	28.4 (26.6, 32.2)	32.2 (28.6, 39.8)	0.051 ^b^
ITMmax	75.94 (74.44, 77.46)	75.6 (74.4, 76.5)	76.6 (74.6, 78.2)	0.25 ^b^
ITM V30	100 (98.88, 100)	99.7 (98.7, 100)	100 (99.5, 100)	0.175 ^b^
ITM V40	86.90 (76.05, 98.65)	84.2 (72.8, 95.3)	93.2 (80.9, 100)	0.141 ^b^
ITM V50	62.8 (24.8)	57.6 (21.8)	68.1 (27)	0.16 ^a^
ITM V60	44 (26.6)	36.7 (21.6)	51.3 (29.5)	0.069 ^a^
ITM V70	27.6 (25.3)	20.7 (18.6)	34.4 (29.4)	0.071 ^a^

^a^ Student’s *t*-test; ^b^ Mann–Whitney *U* test. FOM: floor of the mouth, THM: thyrohyoid muscle, PDS: posterior digastric/stylohyoid muscle complex, LPM: longitudinal pharyngeal muscles, HSG: hyoglossus/styloglossus muscle complex, GGS: genioglossus muscle, ITM: intrinsic tongue muscles.

**Table 3:** Odds ratio of GGS V70 predicting post-radiotherapy penetration or aspiration.

Dosimetric Variable	Crude OR (95% CI)	Adj. OR ^a^ (95% CI)	p_Wald’s test_	p_LR-test_
GGS V70	1.03 (1.01, 1.06)	1.06 (1.03, 1.12)	0.003	<0.001

OR: odds ratio, CI: confidence interval, GGS V70: genioglossus muscle receiving a radiation dose ≥ 70 Gy. ^a^ Adj. OR were adjusted with age, tumor site, pre-treatment Eastern Cooperative Oncology Group performance status, pre-treatment Subjective Global Assessment, tumor stage group, presence of tracheostomy, and time from radiotherapy to swallowing assessment.

## Data Availability

The datasets generated and/or analyzed during the current study are available from the corresponding author on reasonable request.

## Supplementary Materials

The following supporting information can be downloaded at: https://www.mdpi.com/article/10.3390/cancers18040543/s1, Table S1: Balanced variables after propensity score matching.

## Abbreviations

The following abbreviations are used in this manuscript:

CI	Confidence interval
CT	Computed tomography
CTV-HR	High-risk clinical target volume
CTV-IR	Intermediate-risk clinical target volume
DARS	Dysphagia/aspiration-associated structures
FEES	Fiberoptic endoscopic evaluation of swallowing
FOM	Floor of the mouth
GTV	Gross tumor volume
ITM	Intrinsic tongue muscles
LASSO	Least absolute shrinkage and selection operator
LPM	Longitudinal pharyngeal muscles
OR	Odds ratio
PAS	Penetration-Aspiration Scale
PTV	Planning target volume
SGA	Subjective Global Assessment
