# Supplementary material for: Radiation Dose to Swallowing Muscles and Post-Radiotherapy Laryngeal Penetration or Aspiration in Head and Neck Squamous Cell Carcinoma
Source: Cancers (Basel). 2026 Feb 7;18(4):543. doi: 10.3390/cancers18040543 (PMC12938471; doi:10.3390/cancers18040543)
Supplement: Supplementary file 1 [file cancers-18-00543-s001.zip › ESM_1.pdf]

Journal: *Cancers*

Title:

Radiation Dose to Swallowing Muscles and Post-Radiotherapy Laryngeal Penetration or Aspiration in Head and Neck Squamous Cell Carcinoma

Authors:

Thong Chotchutipan[1], Peesit Leelasawatsuk[2], Tiraya Phuengtrakul[2], Jinnatham Apichato[1], Kemmapon Chumchuen[3], Jidapa Bridhikitti[1,*]

Affiliations:

[1] Division of Therapeutic Radiology and Oncology, Department of Radiology, Faculty of Medicine, Prince of Songkla University

[2] Department of Otolaryngology, Faculty of Medicine, Prince of Songkla University

[3] Department of Clinical Research and Medical Data Sciences, Faculty of Medicine, Prince of Songkla University

Corresponding Author:

Jidapa Bridhikitti

Division of Therapeutic Radiology and Oncology, Department of Radiology, Faculty of Medicine, Prince of Songkla University

Tel: +66-74-455-000 ext.1503

Email: cee.jidapa@gmail.com

Table S1. Balanced variables after propensity score matching

Matching variables	Without laryngeal penetration/aspiration	With laryngeal penetration/aspiration	p value	ASMD
Total	22	22		
Age			0.119	
Mean (SD)	55.7 (8.3)	51.2 (10.2)		
Age group			1	< 0.001
≤ 62 years	18 (81.8)	18 (81.8)		
> 62 years	4 (18.2)	4 (18.2)		
Tumor group			1	< 0.001
Nasopharynx	15 (68.2)	15 (68.2)		
Oropharynx	7 (31.8)	7 (31.8)		

SD: standard deviation; ASMD: absolute standardized mean difference
